# Deep Learning for the Classification of Small-Cell and Non-Small-Cell Lung Cancer

**DOI:** 10.3390/cancers12061604

**Published:** 2020-06-17

**Authors:** Mark Kriegsmann, Christian Haag, Cleo-Aron Weis, Georg Steinbuss, Arne Warth, Christiane Zgorzelski, Thomas Muley, Hauke Winter, Martin E. Eichhorn, Florian Eichhorn, Joerg Kriegsmann, Petros Christopolous, Michael Thomas, Mathias Witzens-Harig, Peter Sinn, Moritz von Winterfeld, Claus Peter Heussel, Felix J. F. Herth, Frederick Klauschen, Albrecht Stenzinger, Katharina Kriegsmann

**Affiliations:** 1 Institute of Pathology, Heidelberg University, 69120 Heidelberg, Germany; christian.haag@klinikum-bayreuth.de (C.H.); georg.steinbuss@med.uni-heidelberg.de (G.S.); christiane.zgorzelski@med.uni-heidelberg.de (C.Z.); peter.sinn@med.uni-heidelberg.de (P.S.); moritz.vonwinterfeld@med.uni-heidelberg.de (M.v.W.); albrecht.stenzinger@med.uni-heidelberg.de (A.S.); 2 Translational Lung Research Centre Heidelberg, Member of the German Centre for Lung Research (DZL), 69120 Heidelberg, Germany; thomas.muley@med.uni-heidelberg.de (T.M.); hauke.winter@med.uni-heidelberg.de (H.W.); martin.eichhorn@med.uni-heidelberg.de (M.E.E.); florian.eichhorn@med.uni-heidelberg.de (F.E.); Petros.Christopolous@med.uni-heidelberg.de (P.C.); michael.thomas@med.uni-heidelberg.de (M.T.); hsl19@uni-heidelberg.de (C.P.H.); felix.herth@med.uni-heidelberg.de (F.J.F.H.); 3 Department Hematology, Oncology and Rheumatology, Heidelberg University, 69120 Heidelberg, Germany; 4 Institute of Pathology, University Medical Centre Mannheim, Heidelberg University, 68782 Mannheim, Germany; Cleo-Aron.Weis@medma.uni-heidelberg.de; 5 Institute of Pathology, Cytopathology, and Molecular Pathology, UEGP MVZ Gießen/Wetzlar/Limburg, 65549 Limburg, Germany; Warth@patho-uegp.de; 6 Department of Thoracic Surgery, Thoraxklinik, Heidelberg University, 69126 Heidelberg, Germany; 7 Molecular Pathology Trier, 54296 Trier, Germany; kriegsmann@patho-trier.de; 8 Danube Private University Krems, 3500 Krems, Austria; 9 Department of Thoracic Oncology, Thoraxklinik, Heidelberg University, 69126 Heidelberg, Germany; 10 Medical Faculty Heidelberg University, 69120 Heidelberg, Germany; witzens.harig@yahoo.de; 11 Department of Diagnostic and Interventional Radiology with Nuclear Medicine, Thoraxklinik, Heidelberg University, 69120 Heidelberg, Germany; 12 Department of Diagnostic and Interventional Radiology, Thoraxklinik, Heidelberg University, 69120 Heidelberg, Germany; 13 Department of Pneumology and Critical Care Medicine, Thoraxklinik, Heidelberg University, 69126 Heidelberg, Germany; 14 Institute of Pathology, University Hospital Charité, 10117 Berlin, Germany; frederick.klauschen@charite.de

**Keywords:** artificial intelligence, deep learning, lung cancer, histology, non-small cell lung cancer, small cell lung cancer

## Abstract

Reliable entity subtyping is paramount for therapy stratification in lung cancer. Morphological evaluation remains the basis for entity subtyping and directs the application of additional methods such as immunohistochemistry (IHC). The decision of whether to perform IHC for subtyping is subjective, and access to IHC is not available worldwide. Thus, the application of additional methods to support morphological entity subtyping is desirable. Therefore, the ability of convolutional neuronal networks (CNNs) to classify the most common lung cancer subtypes, pulmonary adenocarcinoma (ADC), pulmonary squamous cell carcinoma (SqCC), and small-cell lung cancer (SCLC), was evaluated. A cohort of 80 ADC, 80 SqCC, 80 SCLC, and 30 skeletal muscle specimens was assembled; slides were scanned; tumor areas were annotated; image patches were extracted; and cases were randomly assigned to a training, validation or test set. Multiple CNN architectures (VGG16, InceptionV3, and InceptionResNetV2) were trained and optimized to classify the four entities. A quality control (QC) metric was established. An optimized InceptionV3 CNN architecture yielded the highest classification accuracy and was used for the classification of the test set. Image patch and patient-based CNN classification results were 95% and 100% in the test set after the application of strict QC. Misclassified cases mainly included ADC and SqCC. The QC metric identified cases that needed further IHC for definite entity subtyping. The study highlights the potential and limitations of CNN image classification models for tumor differentiation.

## 1. Introduction

Based on the GLOBOCAN 2018 produced by the International Agency for Research on Cancer, a database that estimates the incidence and mortality of cancer (including 185 countries and 36 cancers), lung cancer incidence is high and was estimated to be 2.1 million new cases and 1.8 million deaths worldwide, representing 18.4% of all cancer cases [1]. Thus, lung cancer is the most common cancer type among men and the third most common in women worldwide [2]. Smoking is the major risk factor for lung cancer. The 20–fold variation in lung cancer rates in different regions/countries reflects the differences in smoking habits as well as the intensity and type of cigarettes [2,3]. Despite major advances in diagnostics and therapy, mortality remains high, with a five-year tumor-associated mortality of 19%. 

Clinical management highly depends on the histological subtype, as well as immunohistological (IHC) and genetic tumor characteristics [4]. Two major categories are discerned—small-cell lung cancer (SCLC) and non-small-cell lung cancer (NSCLC). The first category constitutes approximately 15%, and the second is responsible for approximately 85% of tumors. The two most common entities in the NSCLC category are pulmonary adenocarcinoma (ADC) and pulmonary squamous cell carcinoma (SqCC), which make up approximately 90% of all NSCLC [5]. Lung cancer is highly heterogeneous, which is reflected by the underlying genetic aberrations that have been detected in the past decades [4,6]. At an advanced clinical stage, individualized therapy highly depends on genetic aberrations involving EGFR, BRAF, ALK, ROS1, RET, etc. [7]. Moreover, the introduction of immune checkpoint and kinase inhibitors has improved prognosis for patients without genetic alterations in these target genes [8,9].

Morphological evaluation of tissue sections remains the basis of histopathological diagnostics and directs the application of additional analyses [10]. In some tumors, the diagnosis can be established on morphology alone, but in a subset of cases, IHC stains are required for definitive diagnosis [11,12]. Currently, the decision of whether to perform IHC is subjective. Moreover, some pathologists can rely on expensive and methodological equipment that allows for liberal use of IHC, while others cannot [13]. Thus, additional methods that support morphological entity subtyping are desirable.

Digital pathology has emerged as an important tool, not only to review histopathological slides on a computer but also to use additional computer-assisted software to support routine diagnostics and research [14,15,16,17]. A prominent example is the evaluation of the intensity and extent of IHC staining that can be assessed by various software applications. It has been shown that proliferative activity can reliably be assessed by computer-assisted evaluation, which in turn supports routine diagnostics in tumors where the proliferation rate plays a major role, such as in neuroendocrine neoplasms [18,19,20]. With these tools, one can extract detailed morphometric information from cells that, after training, allows for automatic detection of tumor and stromal cells [21]. However, as the architectural arrangement of cells is commonly neglected using this approach, different tumor types cannot reliably be differentiated. An alternative approach that allows one to take the architectural pattern into account is the application of convolutional neuronal networks (CNNs) [22,23].

In this study, we applied CNNs and evaluated their capability to classify the most common lung cancer subtypes—namely, SCLC, ADC, and SqCC. Moreover, we developed quality control (QC) measures to objectively detect cases that should be submitted for further evaluation.

## 2. Methods

### 2.1. Patient Cohort, Tissue Microarray Construction, and Scanning of Tissue Slides

A cohort of the three most frequent lung cancer subtypes—SCLC (*n* = 80), ADC (*n* = 80) and SqCC (*n* = 80)—and skeletal muscle (*n* = 30) as a control was assembled from the archive from the Institute of Pathology, University Clinic Heidelberg with the support of the Tissue Biobank of the National Center for Tumor Diseases (NCT). Diagnoses were made according to the 2015 World Health Organization Classification of Tumors of the Lung, Pleura, Thymus, and Heart [12]. In brief, conventional Hematoxlin and Eosin staining as well as immunohistochemistry according to current best practice recommendations were performed [24]. Diagnosis of SCLC was established by morphology as well as through expression of neuroendocrine markers such as synaptophysin, chromogranin and CD56 [25]. Diagnosis of ADC was made if the tumor exhibited growth patterns typical for ADC such as lepidic, acinar, papillary or micropapillary; showed intracytoplasmic reactivity in the Periodic acid–Schiff stain and/or showed immunoreactivity of thyroid transcription factor 1 (TTF-1). Diagnosis of SqCC was rendered if the tumor exhibited intercellular bridges and/or keratinization on morphology, as well as absence of TTF-1 staining and positivity of p40 in more than 50% of tumor cell nuclei using IHC [26]. The study was approved by the local ethics committee (#S-207/2005 and #S315/2020). Formalin-fixed, paraffin-embedded tissue blocks were extracted, and a tissue microarray (TMA) was built as previously described [18,26,27,28]. TMAs were scanned at 400× magnification using a slide scanner (Aperio SC2, Leica Biosystems, Nussloch, Germany).

### 2.2. Tumor Annotation and Image Patch Extraction

**Figure 1:**
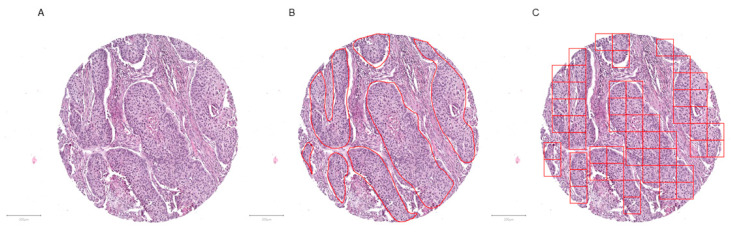
Tumor annotation and generation of image patches. Representative tissue microarray core of a squamous cell carcinoma without (**A**) and with annotation (**B**, red outline), as well as after image patches creation (**C**). The image patches were subsequently saved as .png files. Magnification or scale bars: 200 µm.

**Figure 2:**
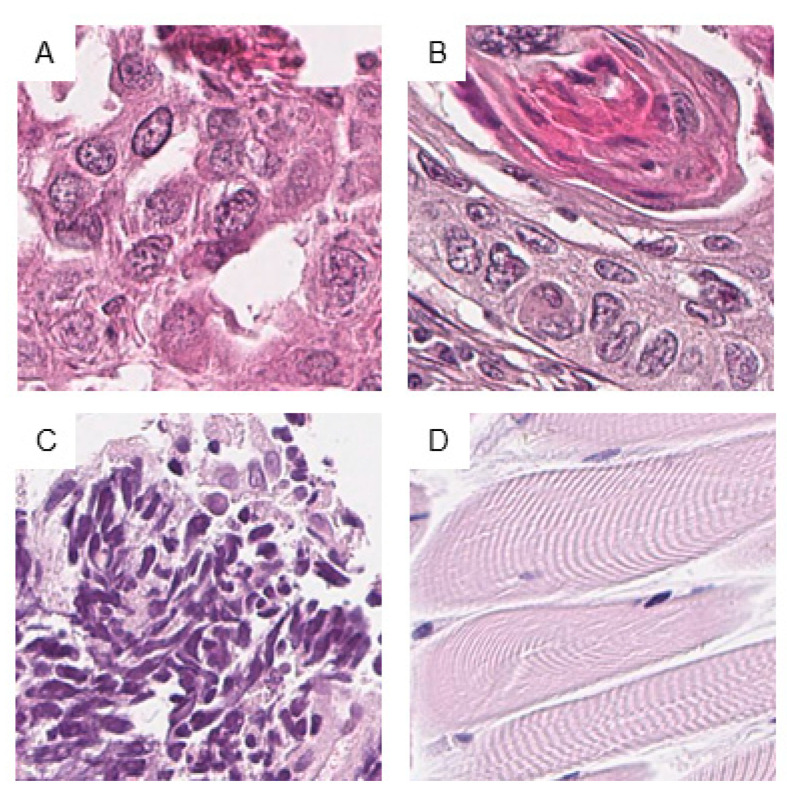
Examples of image patches from annotated areas. One representative image patch from adenocarcinoma (ADC) (**A**), squamous cell carcinoma (SqCC) (**B**), small-cell lung cancer (SCLC) (**C**), and skeletal muscle (**D**) is shown. Magnification or scale bars: each image 100 × 100 µm (395 × 395 px).

Scanned slides were imported into QuPath (v.0.1.2, University of Edinburgh, Edinburgh, UK). Tumor areas of SCLC, ADC, and SqCC as well as from skeletal muscle were annotated by a pathologist (M.K.). Patches 100 × 100 µm (395 × 395 px) in size were generated within QuPath, and the tumor-associated image patches were exported to the local hard drive [21]. To ensure adequate representation of each tumor, the goal of exporting a minimum of 10 patches per patient was set. Representative tumor areas, tumor annotations, generated patches, and extracted patches are displayed (Figure 1 and Figure 2).

### 2.3. Hardware and Software

The following hardware were used for all calculations: Lenovo Workstation p72, CPU Intel(R) Xeon(R) E-2186 M, 2.90 GHz (Intel, Santa Clara, CA, USA), GPU 128 GB DDR4 RAM, GPU NVIDIA Quadro P5200 with Max-Q Design 16 GB RAM (Nvidia, Santa Clara, CA, USA). The following software were used: x64 Windows for Workstations (Microsoft, Redmond, WA, USA), R (v.3.6.2, GNU Affero General Public License v3) and RStudio (v.1.2.5033, GNU Affero General Public License v3) with the packages Keras (v.2.2.5.0), TensorFlow (v.2.0.0) and Tidyverse (v.1.3.0).

### 2.4. Analytical Subsets

To ensure reliable results, image patches were randomly separated into training (60% of patients), validation (20% of patients), and test sets (20% of patients). All image patches from a patient were in one of the sets only. These subsets were not changed during the analyses.

### 2.5. Convolutional Neuronal Network

Our setup using keras and tensorflow in R analytical software allowed us to choose a subset of different network architectures among the hundreds of network architectures available. After a literature review, three different commonly used and previously published CNN architectures were chosen and applied for the analysis. The results were subsequently compared. The CNNs were VGG16, InceptionV3 and InceptionResNetV2 [29,30,31,32,33,34,35]. The size, Top-1 accuracy, Top-5 accuracy on the ImageNet validation dataset, the number of parameters and the depth of VGG16, InceptionV3 and InceptionResNetV2 are as follows: 528 Megabyte (MB), 0.713, 0.901, 138,357,544, 23; 92 MB, 0.779, 0.937, 23,851,784, 159, and 215 MB, 0.803, 0.953, 55,873,736, 572, respectively [36]. The top layer was removed, and an additional network including a flattened layer, a dense layer composed of 256 neurons (ReLu activation function), and an output layer with four classes (Softmax activation function) was put on top of the convolutional base. The optimizer applied was RMSProp with a learning rate of 0.00002. All three network architectures were trained with and without pretrained weights from ImageNet. Different iteration numbers, input image sizes, batch sizes, and dropout rates were evaluated to find a reliable classification model for the training and validation sets. The best model was used to classify the test set.

## 3. Results

### 3.1. Patient Cohort, Annotation, Image Patches Extraction, and Subset Analysis 

**Figure 3:**
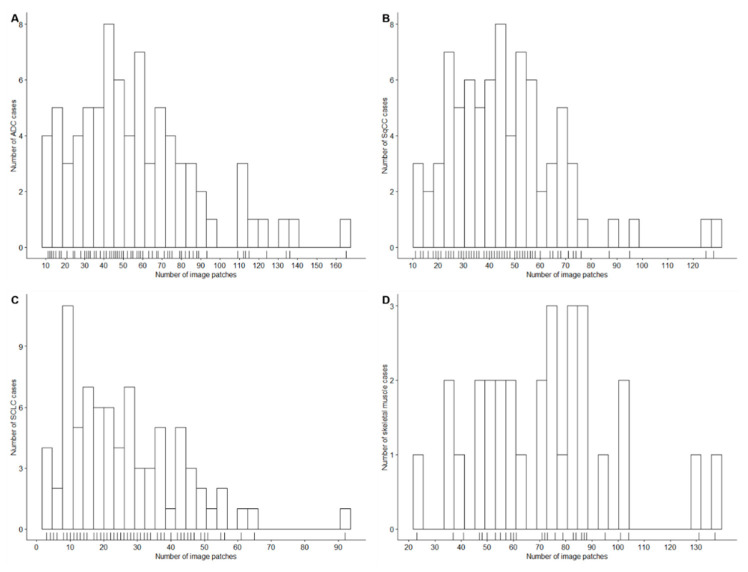
Number of patches according to the tissue type. The histograms show the number of annotated image patches for ADC (**A**), SqCC (**B**), SCLC (**C**), and skeletal muscle (**D**) cases.

Cases from SCLC (*n* = 80), ADC (*n* = 80), SqCC (*n* = 80), and skeletal muscle (*n* = 30) were successfully identified, retrieved, assembled in a TMA, stained, and scanned. Identification of the tumor-containing region resulted in a total of 12,472 extracted 100 × 100 µm (395 × 395 px) image patches. The aim of extracting at least 10 image patches per patient was achieved in all but three SCLC cases, which were still included in the analysis. The number of extracted patches is displayed in Table 1 and Figure 3. Table 1 shows the number of image patches in the training, validation and test sets (60%, 20%, and 20% of patients, respectively) after random patient-based selection.

### 3.2. Convolutional Neuronal Network Selection and Hyperparameter Optimization

Comparison of CNN architectures trained with and without pretrained weights showed a distinct increase in classification accuracy in the former (Table 2A,B). Moreover, overfitting was apparent when more than 20 epochs were trained. Because the classification accuracies of InceptionV3 and InceptionResNetV2 were slightly better in the validation set and the training time was less with the InceptionV3 architecture compared to the InceptionResNetV2 architecture, all other optimization steps were done with the InceptionV3 architecture without pretrained weights and with 20 epochs.

Testing of different input image sizes of 128 × 128 px, 256 × 256 px, and 395 × 395 px revealed a classification accuracy of 83%, 95%, and 93% in the training set and 84%, 89%, and 84% in the validation set, respectively (Table 2C). An input size of 256 × 256 px showed the highest classification accuracy; therefore, this particular image size was chosen for further analysis.

Different batch sizes (8, 16, 32, and 64) were compared. A batch size of 16 had optimal classification accuracy metrics, i.e., 95% in the training set and 89% in the validation set (Table 2D).

As a slight overfitting was noted, different dropout rates (0, 0.1, 0.2, 0.3, 0.4, and 0.5) were evaluated. Compared with the other values, no overfitting was noted with a drop-out rate of 0.5 and a classification accuracy of 88% and 89% in the training and validation sets, respectively (Table 2E).

**Figure A1:**
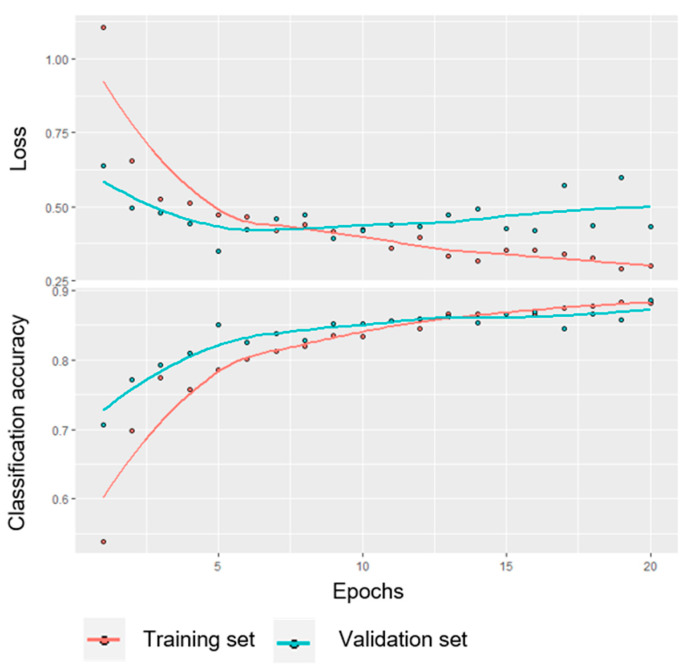
Loss and classification accuracy of the final CNN model.

The variable parameters of the final CNN model and its performance on the training and validation sets are shown in Appendix A Table A1 and Appendix A Figure A1.

The output parameter loss and classification accuracy are shown for the training and validation sets over 20 epochs. The final CNN model parameters were as follows: CNN architecture, InceptionV3; trainable weights, *n* = 192; input image size, 256 × 256 px; image augmentation, yes; batch size, *n* = 16; dropout rate, 0.5; loss function, categorical crossentropy; optimizer, RMSProp; learning rate, 0.00002; and output metrics, accuracy and loss.

### 3.3. Evaluation of the Test Set and Introduction of a Quality Control

**Figure 4:**
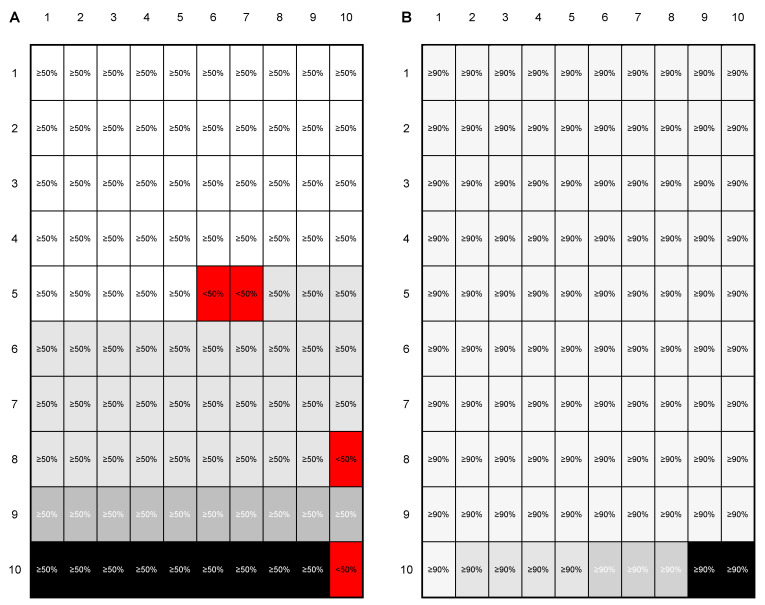
Principle of the introduced image patch and case quality control (QC). To demonstrate the general principle of the introduced QC, two examples—one for 50%/50% image patch/case QC (**A**) and one for 90%/90% image patch/case QC (**B**)—are shown. Given the rationale that a patient case would consist of 100 image patches, 50 and 90 image patches would need a probability of at least 50% (**A**) and 90% (**B**) to fall in a class. In the first example (**A**), 96 image patches have a ≥50% probability to belong to one class (image QC passed in 96 image patches (different shades of grey correspond to the four classes) and failed in four image patches (red)). As <50% of image patches belong to the class with the largest proportion (light grey), the case QC failed. In the second example (**B**) all image patches have a ≥90% probability to belong to one class (image QC passed in all image patches). As >90% of image patches belong to the class with the largest proportion (light grey), the case QC passed.

The final trained CNN model was evaluated on an independent test set. The output of this evaluation was a probability for every single image patch to correspond to one of the four trained classes. However, as an image patch-based classification is not suitable for routine application (i.e., The aim is to classify the whole patient case and not single annotated image patches), two QC parameters were introduced to ensure a high level of classification certainty—(i) a minimum probability for the image patches to fall into one class (image patch QC) and (ii) a minimal proportion of images that need to be classified as one category (case QC). The principle of the two QC categories is shown in Figure 4.

First, the image patch QC was increased from 50% to 90% in 10% increments. With increasing values for the image patch QC, the number of image patches that did not pass the QC increased from 1/2448 (<1%) at an image patch QC of 50% to 386/2448 (16%) at an image patch QC of 90%. Simultaneously, the classification accuracy increased from 89% to 95% in the whole cohort. Most misclassifications were found between ADC and SqCC (Table A1). 

The classification results separated for the whole cohort, for the three lung cancer subtypes, and for the NSCLC subgroup are displayed in Table 3.

Second, case QC was evaluated in combination with image patch QC from 50% to 90% in 10% increments. The results for the whole cohort are displayed in Table 4A. Regardless of the combination of QC values, SCLC and skeletal muscle cases were always correctly classified. Thus, the classification accuracy for the whole cohort was better than that for the NSCLC subgroup. With increasing values for case QC, the number of patients who did not pass increased from 0% to 19% in the whole cohort. The classification results and the number/proportion of cases that did not pass the QC for the three lung cancer subtypes and for the NSCLC subgroup are displayed in Table 4B,C. In the NSCLC subgroup, a classification accuracy of 100% was achieved using image patch and case QCs of 90%. Using these parameters, 31% of cases did not pass QC.

## 4. Discussion

The morphological evaluation of tissue specimens in lung cancer diagnostics is the basis for further molecular testing and therapy stratification [12]. Criteria for additional IHC testing after morphological assessment are subjective. The combination of digital pathology and machine learning has the potential to support this decision process in an objective manner [37,38]. In a previous investigation, the application of deep learning to classify cytological preparations and histological specimens yielded promising results in various cancer types including lung cancer [39,40,41].

In this study, we analyzed whether a CNN-model (InceptionV3 CNN) could be used to differentiate the most common lung cancer subtypes—SCLC, ADC, and SqCC. To check the plausibility of the results, skeletal muscle was also included in the analysis. Histologically, the distinction of skeletal muscle and the three tumor entities is unambiguous. Furthermore, high classification accuracies were expected for the distinction between SCLC and NSCLC, as the cell size is commonly very different [12]. Only in unique cases can separation be difficult by morphology alone, e.g., when the tumor cell count is low, or in specimens with pronounced crush artifacts. The separation between ADC and SqCC is often possible by morphological evaluation alone, but in a subset of cases, only reliable if additional IHC stains are applied. Specifically, poorly differentiated tumors require the use of IHC to identify metastases from extrapulmonary tumors [24,26,28,42,43]. Thus, it was expected that the classification accuracies would be high for skeletal muscle and SCLC but rather intermediate for ADC and SqCC.

In this study, we used a TMA to extract the image patches for several reasons. First, the tumor-containing area of each patient is comparable [18]. Second, the number of extracted image patches is limited, which saves computational resources. Third, the scan time and hard drive space is lower, and fourth, more tumors can be annotated at the same time by using whole slide annotations. Moreover, a TMA is suitable to mimic the biopsy situation [44]. Once the algorithm is trained, it can be applied to image patches extracted from TMAs, biopsies or resection specimens and therefore is in principle applicable in the routine setting.

The creation of image patches from a scanned image is necessary, as CNN can process only limited image sizes [45]. The separation of 60%, 20%, and 20% for the training, validation, and test sets was arbitrary, and there is currently no established gold standard [38,46,47,48]. A higher proportion of cases in the training set would result in a more robust model, but the data in the validation and test cohort would possibly not be representative. Nonetheless, separation into the three sets is mandatory, as during hyperparameter tuning, information from the training set migrates into the validation set. Thus, the capacity of the model must be tested on a separate test set.

In the past, various CNN architectures and modifications have been developed, and some show a high classification accuracy in the ImageNet dataset [49,50,51]. However, as the newer CNN architectures were not (yet) implemented in the software that was used here, we choose CNN architectures that were previously used to classify image data and were available in our software. Because it has been shown that the pretrained weights from the ImageNet dataset can also be used to efficiently classify new images, the CNN architectures were evaluated both with and without pretrained weights [38,46]. However, as the classification accuracy was distinctly lower with pretrained weights, we choose to use the CNN architectures without pretrained weights [38]. There is no established standard for the optimization process of a CNN model, but all parameters used in this study were within the range of reported variations [52,53,54,55].

The final model was robust and reached an image patch classification accuracy of 88% in the training as well as in the validation set which is comparable to previous studies using histological images [56,57] Mainly ADC and SqCC were misclassified, as expected. For a routine application of a CNN for entity subtyping, a classification based on patients is much more meaningful. Therefore, the entity was defined by the proportion of image patches that were most common. As expected, a higher value for the QC resulted in a higher proportion of cases with a failed QC. Irrespective of the evaluated subset (whole cohort, three lung cancer subtypes or NSCLC cases), the classification accuracy increased to 100% using image patch and case QC cutoffs of 90%. For the ADC and SqCC subgroups, 31% of patients did not meet the QC criteria using image patch and case QC cutoffs of 90%. Thus, the CNN classification model and the subsequent application of QC measures allowed us to objectively identify cases that needed further IHC evaluation for definite entity subtyping.

The limitations of our study are the sample size, the number of extracted image patches in some cases, the number of included entities and the process for hyperparameter tuning. Herein, we examined 80 cases per lung cancer entity. Based on the random separation into training, validation and test sets, only 48 tumors were included in the training set. ADC and SqCC may be morphologically very different, and many variants are recognized in the current World Health Organization classification [12]. Furthermore, there may be mixed tumors such as SCLC combined with large cell neuroendocrine tumors or adenosquamous carcinomas [58,59]. Based on the broad biological variation, it becomes clear that the limited number of cases and extracted image patches per patient can only display a fraction of the possible morphological spectrum. Moreover, it is apparent that mixed tumors are a particular challenge for CNN-based classifications. Our model was trained to detect only the three most common lung cancer entities. Therefore, it cannot be expected that the CNN will reliably classify entities that were not trained, including other pulmonary or extrapulmonary tumors. Moreover, a small number of tumor cells per image patch may be a limiting factor and the minimal number of tumor cells needed for a reliable result is currently not clear. Thus, additional QC measures merit further investigation. Based on the abovementioned statements, the application of CNN for tumor classification must always be conducted under the supervision of a pathologist to avoid misdiagnosis and potentially harmful consequences for patients. Finally, hyperparameter optimization was conducted sequentially. As not all possible hyperparameter combinations were tested, there is a possibility that there is an even better combination of hyperparameters. However, as hyperparameter tuning in our study resulted only in minor improvements, it was assumed that the influence of a better combination of hyperparameters would be minimal.

## 5. Conclusions

In summary, we trained and optimized a CNN model to reliably classify the three most common lung cancer subtypes. Moreover, we established QC measures to objectively identify cases that need further IHC validation for reliable entity subtyping. Our results highlight the potential and limitations of CNN image classification models for morphology-based tumor classification.

## Figures and Tables

**Table 1:** Descriptive statistics of annotated image patches and analysis subsets.

Tissue Type	ADC	SqCC	SCLC	Skeletal Muscle	Overall Sum
**Overall Analysis set, 100% of Cases**					
Cases, *n*	80	80	80	30	
Image patches, *n*					
Sum	4505	3695	2075	2152	12,427
Minimum	11	11	3	23	
Maximum	165	128	92	137	
Mean	56	46	26	72	
Median	51	43	23	73	
**Training Set, 60% of Cases**					
Cases, *n*	48	48	49	18	
Image patches, *n*					
Sum	2686	2108	1253	1298	7345
Minimum	11	13	3	37	
Maximum	165	95	92	131	
Mean	56	44	26	72	
Median	54	43	22	73	
**Validation Set, 20% of Cases**					
Cases, *n*	16	16	15	6	
Image patches, *n*					
Sum	871	845	437	479	2632
Minimum	15	11	4	37	
Maximum	136	128	65	137	
Mean	54	53	29	80	
Median	46	48	28	72	
**Test Set, 20% of Cases**					
Cases, *n*	16	16	16	6	
Image patches, *n*					
Sum	948	742	385	375	2450 *
Minimum	13	19	4	23	
Maximum	115	76	56	87	
Mean	59	46	24	63	
Median	55	41	23	70	

* Two image patches were removed at random to ensure divisibility by the batch size of 16 (16 × 153 = 2448). ADC: adenocarcinoma; SqCC: squamous cell carcinoma; SCLC: small-cell lung cancer.

**Table 2:** Classification accuracy of different convolutional neuronal network (CNN) models during the optimization process.

A. CNN Models with Pretrained Weights on the ImageNet Dataset										
CNN	VGG16				InceptionV3			InceptionResNetV2		
Epochs, *n*	20		50		20	50		20		50
Training set	81%		82%		68%	70%		72%		74%
Validation set	81%		81%		59%	64%		62%		60%
**B. CNN Models with Weights Trained on the Training Set**										
**CNN**	**VGG16**				**InceptionV3**			**InceptionResNetV2**		
Epochs, *n*	20		50		20	50		20		50
Training set	88%		91%		83%	88%		87%		89%
Validation set	83%		86%		86%	85%		85%		84%
**C. Different Image Input Sizes**										
**Input size, px**	**128 × 128**				**256 × 256**			**395 × 395**		
Epochs, *n*	20				20			20		
Training set	83%				95%			93%		
Validation set	84%				89%			84%		
**D. Different Batch Sizes**										
**Batch size, *n***		**8**		**16**			**32**		**64**	
Epochs, *n*		20		20			20		20	
Training set		84%		95%			94%		96%	
Validation set		88%		89%			87%		89%	
**E. Different Dropout Rates**										
**Dropout rate**	**0**		**0.1**		**0.2**	**0.3**		**0.4**		**0.5**
Epochs, *n*	20		20		20	20		20		20
Training set	95%		89%		89%	88%		89%		88%
Validation set	89%		86%		84%	86%		86%		89%

CNN: Convolutional Neural Network.

**Table 3:** Proportion of image patches with failed QC and classification accuracy according to image patch QC.

Image Patch QC Value	Image Patches with Failed QC (*n*)	Proportion of Image Patches with Failed QC (%)	Classification Accuracy of ADC, SqCC, SCLC, Skeletal Muscle Image Patches (%)	Classification Accuracy of ADC, SqCC, SCLC Image Patches (%)	Classification Accuracy of ADC, SqCC Image Patches (%)
50%	1	0.04	89	87	85
60%	79	3	91	89	87
70%	150	6	92	90	89
80%	255	10	93	92	90
90%	389	16	95	94	92

The proportion of image patches with failed QC was calculated in all ADC, SqCC, SCLC, and skeletal muscle image patches of the test set (n_overall_ = 2448).

**Table 4:** Classification accuracy and proportion of cases in which QC failed.

Case QC Value		50%		60%		70%		80%		90%	
Parameter		CA (%)	QC Failed (%)	CA (%)	QC Failed (%)	CA (%)	QC Failed (%)	CA (%)	QC Failed (%)	CA (%)	QC Failed (%)
**A. ADC, SqCC, SCLC, and Skeletal Muscle Cases**											
**Image patch QC value**	50%	94	0	96	6	98	11	100	19	100	24
	60%	94	0	98	4	98	9	98	17	100	22
	70%	94	0	98	6	98	9	98	15	100	20
	80%	94	0	98	4	98	9	98	13	100	19
	90%	96	0	98	4	98	7	98	9	100	19
**B. ADC, SqCC, and SCLC Cases**											
**Image patch QC value**	50%	94	0	96	6	98	13	100	21	100	27
	60%	94	0	98	4	98	10	97	19	100	25
	70%	94	0	98	6	98	10	98	17	100	23
	80%	94	0	98	4	98	10	98	15	100	21
	90%	96	0	98	4	98	8	98	10	100	21
**C. ADC and SqCC Cases**											
**Image patch QC value**	50%	91	0	93	9	96	19	100	31	100	41
	60%	91	0	97	6	96	16	96	28	100	38
	70%	91	0	97	9	96	16	96	25	100	34
	80%	91	0	97	6	96	16	96	22	100	31
	90%	94	0	97	6	96	13	96	16	100	31

The proportion of cases with failed QC was calculated in all ADC (*n* = 16), SqCC (*n* = 16), SCLC (*n* = 16) and skeletal muscle (*n* = 6) cases of the test set (*n* = 54).

## Appendix A

**Table A1:** True and predicted diagnoses of test set image patches according to different image patch QC values.

Image Patch QC Value	True Diagnosis	Predicted Diagnosis				Image Patch QC Failed
		ADC	SqCC	SCLC	Skeletal Muscle	
50%						
	ADC	798	137	12	0	1
	SqCC	116	624	0	0	0
	SCLC	0	0	385	0	0
	Skeletal muscle	0	0	0	375	0
60%						
	ADC	776	109	12	0	51
	SqCC	101	611	0	0	28
	SCLC	0	0	385	0	0
	Skeletal muscle	0	0	0	375	0
70%						
	ADC	753	86	12	0	97
	SqCC	89	598	0	0	53
	SCLC	0	0	385	0	0
	Skeletal muscle	0	0	0	375	0
80%						
	ADC	721	66	11	0	150
	SqCC	71	564	0	0	105
	SCLC	0	0	385	0	0
	Skeletal muscle	0	0	0	375	0
90%						
	ADC	672	46	10	0	220
	SqCC	51	523	0	0	166
	SCLC	0	0	385	0	0
	Skeletal muscle	0	0	0	375	0

The values represent absolute numbers.
